# Preparation and *in vitro* performance evaluation of resveratrol for oral self-microemulsion

**DOI:** 10.1371/journal.pone.0214544

**Published:** 2019-04-16

**Authors:** Hongwei Tang, Shu Xiang, Xiangzhou Li, Jun Zhou, Chuntao Kuang

**Affiliations:** 1 College of Materials Science and Engineering, Central South University of Forestry & Technology, Changsha, China; 2 State Key Laboratory of Ecological Applied Technology in Forest Area of South China, Changsha, China; Brandeis University, UNITED STATES

## Abstract

The purpose of this study was to improve the solubility of resveratrol (Res) by a self-microemulsifying drug-delivery system (SMEDDS). Through a solubility experiment, the pseudoternary phase diagram and ternary phase diagram were used to optimize the Res SMEDDS formula. The optimum formulation consisted of 5% IPM, 20% PEG400, and 65% Cremophor RH40. The water solubility, stability, *in vitro* release and antioxidant activity of the Res SMEDDS were characterized. The Res solubility in the SMEDDS was at least 1,000 times compared to that in water. The average droplet size of the microemulsion was 28.00±1.67 nm and uniform distribution. The Res SMEDDS should be stored at low temperature and in the dark to avoid light conditions. Res SMEDDS was able to improve the *in vitro* release rate of Res, and the *in vitro* release of Res from Res SMEDDS was significantly faster that of Res powder and unaffected by pH value of media. Antioxidant assays showed that antioxidant activities of Res in Res SMEDDS were unaffected compared to Res powder. Cytotoxicity study indicated that Res SMEDDS at the concentration of less than 100 μM was safe. These results demonstrated the potential use of Res SMEDDS for oral administration of Res.

## Introduction

Resveratrol (Res), a natural polyphenol derived from plants, such as Polygonum cuspidatum, grape, peanut and mulberry[1–4], has a wide range of pharmacological activities, including anticancer, antioxidant, anti-inflammatory and antineuralgic[5–9], thus, it has attracted the attention of researchers. However, Res is a class II drug in the Biopharmaceutics Classification System (BCS) with poor water solubility (0.03 mg/mL) and high permeability.[10–12] Accordingly, improving the solubility of Res is a top priority.

Self-microemulsifying drug delivery systems (SMEDDS) are isotropic mixtures of oils, hydrophilic emulsifiers and co-emulsifiers. SMEDDS possess thermodynamic stability and are spontaneously emulsified into droplets of size in the range of 10–100 nm under slight stirring[13]. The SMEDDS is used for the improvement of the bioavailability of poorly soluble drugs based on high stability, low viscosity and simple preparation[14–16].

There are many studies on the use of SMEDDS as carrier of poorly soluble drugs. Compared with the total flavones of *Hippophaë rhamnoides* L. (TFH), the TFH SMEDDS significantly enhances the solubility of the TFH up to 530 times in water, and its relative bioavailability is dramatically improved 3.09 times[17]. Wu X et al. also reported that the SMEDDS improved the water solubility of curcumin, increasing the relative oral bioavailability of the SMEDDS by 12.13 times compared with pure curcumin[18]. Compared with pure Res, the Res SMEDDS exhibited excellent antioxidant activity and less toxicity[15].

The aim of this study is to develop an oral Res SMEDDS with excellent performance. The solubility test and pseudoternary phase diagram were used to select the composition of the SMEDDS. The water solubility, stability, release performance and antioxidant activity of the Res SMEDDS were assessed. Additionally, the particle size and morphology of the Res SMEDDS were investigated by laser particle size analysis and transmission electron microscopy (TEM).

## Materials and methods

### Reagents and materials

Res (98% *w*/*w*) was obtained from Huayuan County Hengyuan Plants Biochemical Co., Ltd. (Huayuan, Hunan, China). Corn germ oil was purchased from China National Cereals, Oils and Foodstuffs Co. Ltd. (Beijing, China). Isopropyl myristate (IPM), ethyl oleate, Tween 20, Tween 60, Tween 80, hydrogenated castor oil polyoxyethylene ether (Cremophor RH40), castor oil polyoxyethylene ether (Cremophor EL), glycerol, propylene glycol, polyethylene glycol 400 (PEG400), and anhydrous ethanol were purchased from Sinopharm Chemical Reagent Co., Ltd. (Shanghai, China). Sodium dihydrogen phosphate, disodium hydrogen phosphate, and hydrochloric used to prepare the *in vitro* release media were obtained from Sinopharm Chemical Reagent Co., Ltd.. 2,2-Diphenyl-1-picrylhydrazyl (97%w/w, DPPH·) and 2,2''-azino-bis (98%w/w, ABTS^+^·) used to assess *in vitro* antioxidant activity of Res SMEDDS, were purchased from TCI Chemical Industry Development Co., Ltd. (Shanghai, China) and Shanghai Lanji Technology Development Co., Ltd (Shanghai, China), PC12 cells were obtained from Changsha Auragene Biotechnology Co., Ltd (Changsha, China), DMEM medium was purchased from Hyclone(Logan,UT,USA) and foetal bovine serum was from Gemini (Calabasas,California,USA), Penicillin/streptomycin and DMSO were purchased from MP Biomedicals,LLC (Santa Ana, California, USA), MTT kit were purchased Sangon Biotech (Shanghai) Co.,Ltd (Shanghai, China). respectively. All the above reagents were analytical grade or better.

The morphology of the Res SMEDDS was examined on a S-3400N Biomedical scanning electron microscope (Hitachi, Ltd., Tokyo, Japan). the particle size and polydispersity index (PDI) of the Res SMEEDS were determined with a Mastersizer 2000 Laser Particle Size Analyzer (Malvern Instruments Co., Ltd., Malvern, UK). The TU-1901 Ultraviolet Spectrophotometer (Beijing Persee General Instrument Co., Ltd., Beijing, China) was used to determine the resveratrol content. The *in vitro* release experiment was performed on a RC-3 dissolution instrument (Xin Tian Guang Analytical Instrument Technology Co., Ltd., Tianjin, China). TG16-WS high-speed centrifuge (General Instrumentation Co., Ltd., Changsha, China) was used to perform the centrifugation steps during the preparation of Res.

### Solubility Study of Resveratrol (Res) in Various Oils, emulsifiers, and Co-emulsifiers

The solubility of Res was measured in various oils, emulsifiers, and co-emulsifiers. An excessive amount of Res powder was added to 10.0 g of various oils (isopropyl myristate, corn germ oil, ethyl oleate), emulsifier (Tween 20, Tween 60, Cremophor EL, Cremophor RH), and co-emulsifier (glycerol, propylene glycol, PEG400, anhydrous ethanol). After stirred with a magnetic stirrer for 30 minutes, the mixture was shaken for 48 h at 37°C. Then the mixture was centrifuged at 10,000 r/min for 10 min, and the supernatant was filtered with 0.45-μm filter. The concentration of Res was determined by UV spectrophotometry at 305nm.

### Determination and optimization of the self-microemulsion formula

#### Compatibility of oil phase with emulsifier

The oil and emulsifier, at a mass ratio of 2:3, were mixed at 37°C, then, 0.2 mL of the mixture was added dropwise into 20 mL of deionized water with stirring at 100 r.min^-1^ at 37°C.The performance of the formulations was visually assessed by the following grading system[19]: (A) Microemulsion which was clear or slightly bluish in appearance. (B) Slightly less clear emulsion which had a bluish white appearance. (C) A bright white emulsion. (D) A dull, greyish white emulsion with a slightly oily appearance. (E) A formulation which exhibited either poor or minimal emulsification with large oil droplets present on the surface. Based on the above conditions, suitable oils, emulsifiers and co-emulsifiers were preliminary screened.

#### Construction of pseudo-ternary phase diagram

The ability of different emulsifiers and co-emulsifiers to form oil-in-water (O/W) microemulsions was investigated. Briefly, the emulsifier and the co-emulsifier with a mass ratio of 1:1 were uniformly mixed, then, the oil phase and the emulsifier/co-emulsifier mixture were mixed at various ratios (*w*/*w*) ranging from 9:1, 8:2, 3:7, 4:6 …2:8, 1:9. The mixture was titrated with deionized water at 37°C with stirring. The pseudoternary phase diagram was plotted according to the mass percentage of each component at the critical point of the O/W microemulsion. Ultimately, the components with larger O/W microemulsion were selected for the optimum formulation.

#### Formula optimization

The ternary phase diagrams of the mixtures of oil, emulsifier, and co-emulsifier were prepared. Briefly, according to the pseudoternary phase diagram results, the optimal oil, emulsifier, and co-emulsifier at a certain ratio were mixed in tubes and kept for 4 h. Then, 0.2 mL of the unstratified mixture was added to 20 mL of water (37°C) with stirring at 100 r.min^-1^, and the emulsion time and final appearance were noted. The boundaries of the SMEDDS regions in the phase diagrams were determined by connecting the points representing the formation of the microemulsion[20, 21], with the formulation based on the ternary phase diagram.

### Characterization of the SMEDDS

#### Appearance

The Res SMEDDS was serially tenfold diluted (10, 100, and 1000 times) with deionized water and then the concentration was assessed by comparing with the appearance of the same concentration of Res solution.

#### Morphology, size distribution and zeta potential

The blank SMEDDS and Res SMEDDS was diluted 100 times with deionized water to obtain the emulsion, and the particle size distribution was determined immediately by a Malvern laser particle size analyzer. TEM analysis was performed to determine the microstructure of the Res emulsion with the method reported by Chen Y et al[15].

### Stability study

The light stability and thermal stability of the Res SMEDDS were studied with reference to the relevant provisions of Appendix XIXC “Stability Testing of Drug Substances and Products” of the Chinese Pharmacopoeia (2015 edition).

#### *In vitro* drug release

*In vitro* release of the Res SMEDDS and Res was tested by the method of Pineros et al[18] with some modifications. Briefly, a 900-mL solution composed of hydrochloric acid solution (pH = 1.2), phosphate buffer (pH = 6.8) and phosphate buffer (pH = 7.4) was used as dissolution medium with stirring at 50 r/min for 37°C. An aliquot (5 mL) of the sample was collected at 5, 10, 20, 30, 45, and 60 min. At the same time, an equivalent volume (5 mL) of fresh dissolution medium was added to compensate for the removed volume. The sample was filtered through a 0.45-μm filter and the concentration of Res was measured by UV spectrophotometry. The release of the Res SMEEDS and Res at different pH values was examined with the same quantity of drug.

#### DPPH Free radical scavenging experiment

The DPPH free radical scavenging of the Res SMEEDS and Res was analyzed by the method of Pápay et al. with slight modifications[22]. Briefly, a 79-mg/L DPPH· ethanol solution was freshly prepared and protected from light. Solutions of Res SMEDDS and Res at various concentrations (200, 400, 600, 800, and 1000 μg/L) were prepared in ethanol, and 0.5 mL of each of these samples with different concentrations was mixed with 9.5 mL of DPPH· solution, and the mixture was shaken at 37°C for 1 h in the dark. The absorbance at 517 nm was determined with a UV/Vis spectrophotometer, the DPPH· scavenging rate was calculated according to the following equation (Eq 1):

$$Scavenging\;Rate/\%=\frac{A_{Blank}-A_{Samples}}{A_{Blank}}\times 100\%$$ (1)

Where A_blank_ is the absorbance of the DPPH· solution, and A_Sample_ is the absorbance of the sample. All measurements were performed in triplicate.

#### ABTS Free radical scavenging experiment

The free radical scavenging activity was measured by the ABTS^+·^ method as described previously with slight modifications[23]. The ABTS stock solution was prepared by dissolving it in water to a concentration of 3.84g/L. The ABTS free radical (ABTS^+·^) was prepared by reacting the ABTS stock solution and 1.34 g/L potassium persulfate at a volume ratio of 1:1, and the mixture was stored in the dark at room temperature for 12 h before use. The blue-green ABTS^+·^ solution was adjusted to an absorbance of 0.70±0.02 at 734 nm with additional water. Solutions of Res SMEDDS and Res at concentrations (40, 60, 80, 100, and 200 μg/L) were prepared in ethanol, 0.5 mL of each of these samples with different concentrations was added to 9.5mL of ABTS^+·^solution, and the mixture was shaken at 37°C for 1h in the dark. The absorbance was determined at 734 nm on a UV/Vis spectrophotometer, the ABTS^+·^ scavenging rate is calculated according to Eq 1.

$$Scavenging\;Rate/\%=\frac{A_{Blank}-A_{Samples}}{A_{Blank}}\times 100\%$$ (2)

Where A_blank_ is the absorbance of the ABTS^+·^ solution, and A_Sample_ is the absorbance of the sample. All measurements were performed in triplicate.

#### Cytotoxicity assays

The cytotoxicity of Res SMEDDS and Res was determined by a MTT kit according to the manufacturer’s instructions. Briefly, 5×10^4^ cell/mL of PC12 cells suspended in DMEM SH30022.01 medium (100μL) containing 10% fetal bovine serum and 5% FBS were seeded into 96-well plates. 100μL containing 10% MTT solution was added to each well, and the cells were incubated at 37°C for 4 h. Carefully remove the culture solution in the well and add 100 μL of DMSO to each well to dissolve the crystals. The absorbance was then measured at 570 nm. The cytotoxicity of the Res SMEDDS and Res and was proceed through indirect contact testing according to MTT assay of ISO 10993-5-2009, Calculate the relative growth rate (RGR) of the sample according to Eq 3, and the cytotoxicity fraction of the Res SMEDDS and Res was evaluated according to Table 1.

$$RGR/\%=\frac{OD_{T\mathrm{est}}}{OD_{C\mathrm{ontrol}}}\times 100\%$$ (3)

Where OD_Test_ is the absorbance of the experimental group, and OD_Control_ is the absorbance of the control group. All measurements were performed in four times.

**Table 1:** Cytotoxicity grades and corresponding relative growth rates.

Grades	0	1	2	3	4	5
RGR/%	≥100	75~99	50~74	25~49	1~24	0

Grades 0 and 1 were considered to be non-cytotoxic, grade 2 was mildly cytotoxic, grades 3 and 4 were moderately cytotoxic, and grade 5 was markedly cytotoxic.

## Results and discussion

### Preliminary screening of the SMEEDS formula components

The SMEEDS excipient should have excellent solubilization capacity for the drug, which is essential for allowing presentation of the drug in SMEEDS, and excellent compatibility between emulsifiers and co-emulsifiers was found to be beneficial to the formation of small particle size emulsion[18, 19].

**Fig 1:**
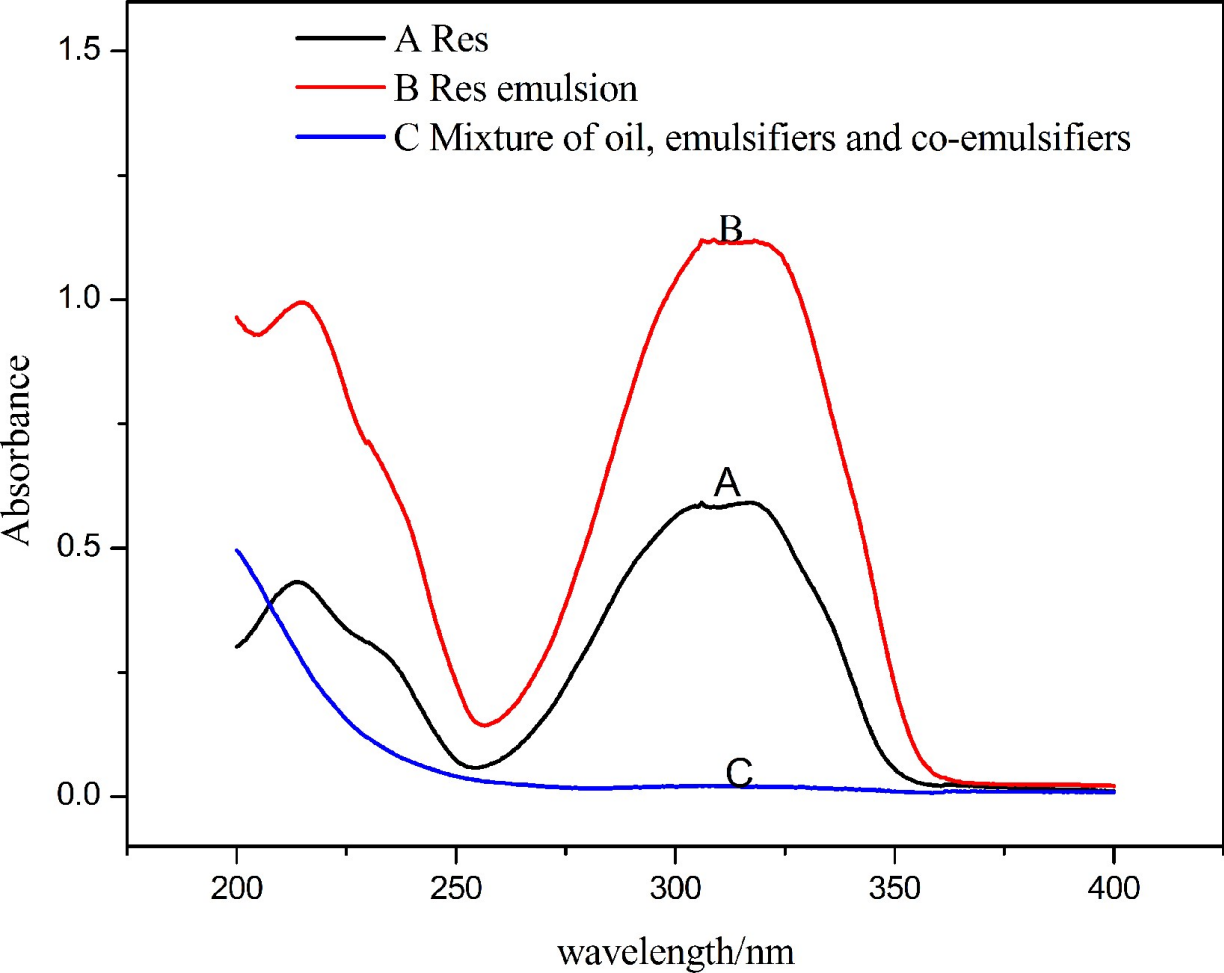
Ultraviolet spectrum. (A)UV spectra of Res.(B) UV spectra of Res emulsion. (C) UV spectra of the mixture of oil, emulsifier, and co-emulsifier.

Compared with the mixture of oil, emulsifier, and co-emulsifier, Res exhibited a UV typical maximal absorption peak at 305 nm (Fig 1).The solubility of Res in the oil phases, emulsifier and co-emulsifier are listed in Table 2. There was no significant difference in the solubility of Res in the three oil phases measured *(P>0*.*05)*, but IPM was more compatible with the emulsifiers than corn germ oil and ethyl oleate (Table 3). Accordingly, IPM was selected as the oil phase. Compared with Tween 20 and Tween 60, Cremophor EL and Cremophor RH40 have a stronger emulsifying capacity due to their larger number of ethylene oxide (EO), thus they are more compatible with the three oil phases[24]. Besides, Res has high solubility in Cremophor EL and Cremophor RH40, therefore, Cremophor EL and Cremophor RH40 were chosen as the alternative emulsifiers for subsequent comparisons. Among the co-emulsifiers, Res has relatively high solubility in anhydrous ethanol, PEG400 and propylene glycol, therefore, they were selected as preliminary co-emulsifiers.

**Table 2:** Solubility of Resveratrol (Res) in different vehicles (37°C).

Excipient	Vehicles	Solubility (mg/mL)
Oil	IPM	0.38±0.08
	Corn germ oil	0.28±0.08
	Ethyl oleate	0.24±0.06
Emulsifier	Tween 20	248.38±4.52
	Tween 60	105.16±3.75
	Cremophor EL	190.53±5.23
	Cremophor RH40	148.10±4.89
Co-emulsifier	Glycerin	3.83±0.38
	Propylene glycol	63.96±3.25
	PEG400	139.95±4.51
	Anhydrous ethanol	129.86±4.82

**Table 3:** Compatibility of the oil phase and emulsifier.

	Tween 20	Tween 60	Cremophor EL	Cremophor RH40
IPM	A	A	A	A
Corn germ oil	E	E	C	C
Ethyl oleate	C	A	A	A

A,Microemulsion which was clear or slightly bluish in appearance. B,Slightly less clear emulsion which had a bluish white appearance. C, A bright white emulsion. D, A dull, greyish white emulsion with a slightly oily appearance. E, A formulation which exhibited either poor or minimal emulsification with large oil droplets present on the surface.

### Screening of emulsifiers and co-emulsifiers

**Fig 2:**
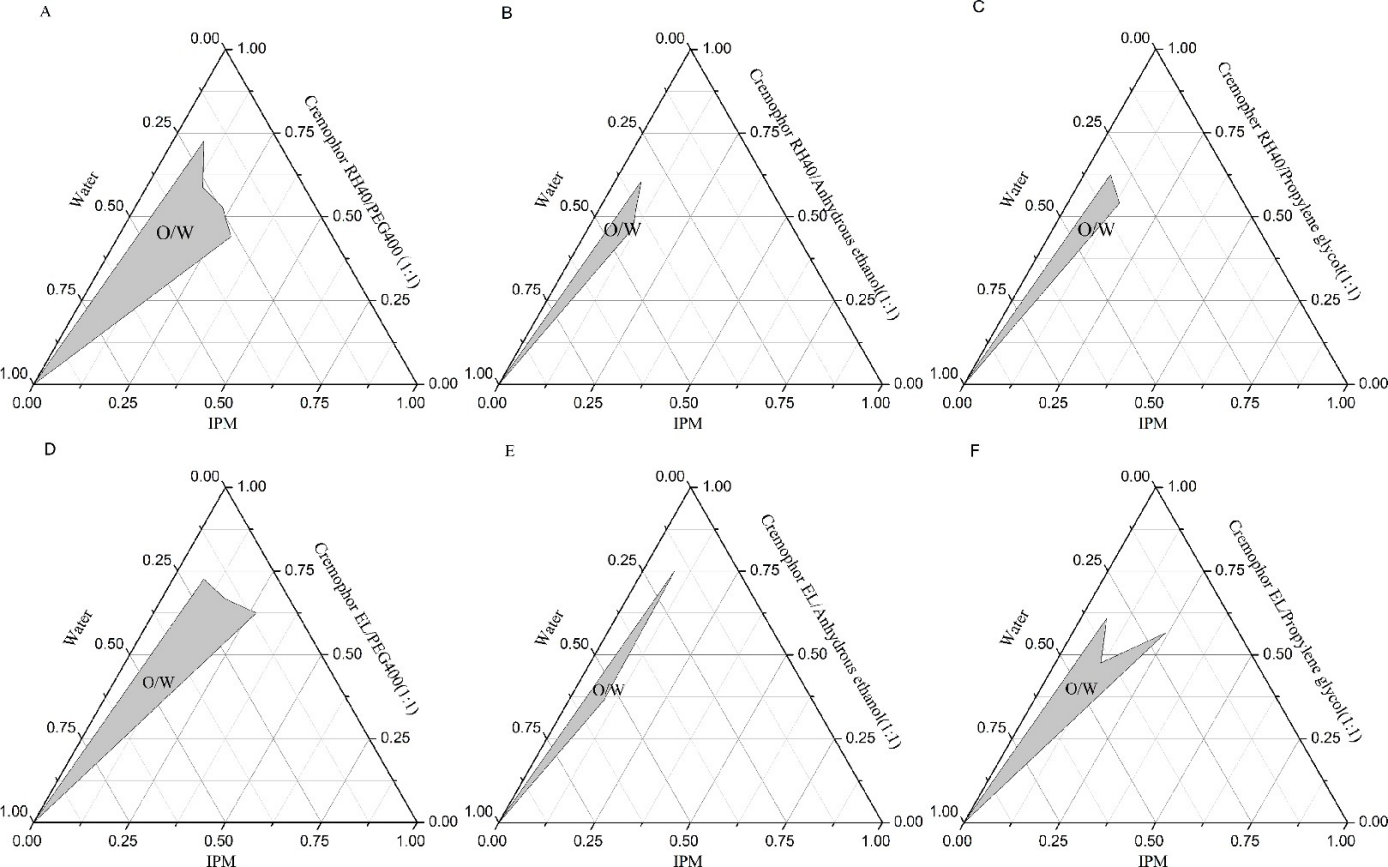
Pseudoternary phase diagrams for the formulas (The shaded region represents the O/W microemulsion region). (A) IPM/Cremophor RH40/PEG400 system; (B) IPM/Cremophor RH40/ Anhydrous ethanol system;(C) IPM/Cremophor RH40/ Propylene glycol; (D) IPM/Cremophor EL/PEG400 system; €IPM/Cremophor EL/ Anhydrous ethanol system; (F) IPM/Cremophor EL/ Propylene glycol.

The co-emulsifier can increase the fluidity of the oil-water interface film and reduce the surface tension of the oil-water interface[25], which is beneficial to the formation of the microemulsion, the larger the microemulsion region is, the stronger the emulsifying ability is[26]. When IPM was used as the oil phase, the pseudoternary phase diagram of the different co-emulsifiers and emulsifiers are shown in Fig 2. Cremophor RH40 or Cremophor EL used as emulsifier, and PEG400 used as co-emulsifier (Fig 2A and 2D) with moderate molecular weight exhibited excellent ability to assist the emulsification of microemulsions compared to shorter anhydrous ethanol (Fig 2B and 2E) and propylene glycol (Fig 2C and 2F), Therefore, PEG400 was chosen as co-emulsifier.

Although both Cremophor RH40 (hydrophilic-lipophilic balance (HLB) = 14–16) and Cremophor EL (HLB = 12–14) could form O/W microemulsions with the same co-emulsifier (PEG400), there was quite a difference in their emulsification ability. As shown in Fig 2A and 2D, the O/W microemulsion region with Cremophor RH40 (HLB = 14–16) is larger than that with Cremophor EL (HLB = 12–14), because the larger the HLB value of the emulsifier is, the stronger the emulsifying capacity is. Moreover, the toxicity of Cremophor RH40 is smaller[27]. Therefore, Cremophor RH40 was selected as the emulsifier. Based on the above data, the oil phase, emulsifier and co-emulsifier of SMEDDS formulation were IPM, Cremopher RH40 and PEG400, respectively.

### Self-microemulsion phase diagram construction and formula optimization

**Fig 3:**
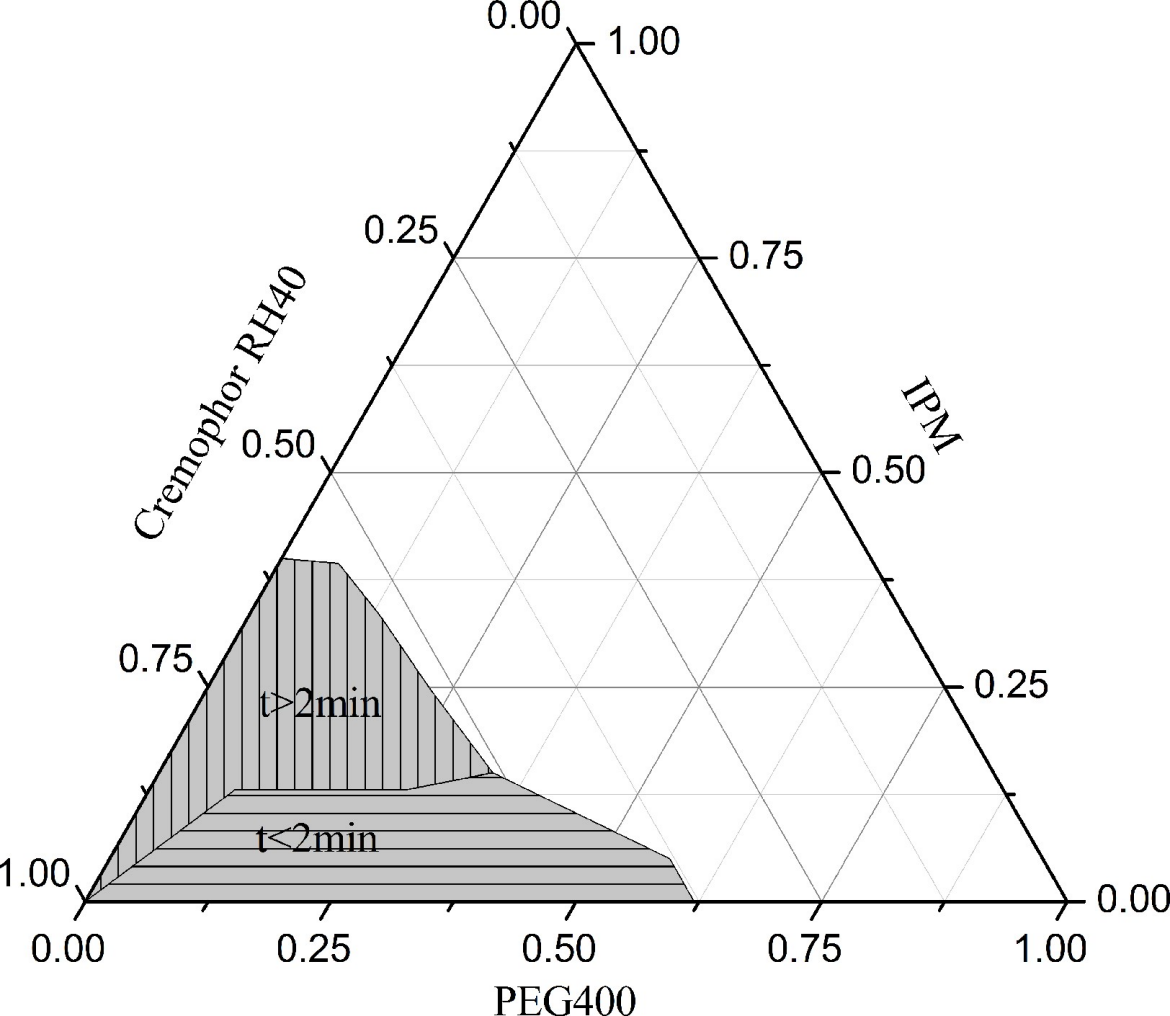
Ternary phase diagram of the SMEDDS (The shaded region represents the SMEEDS, the area region along the horizontal line represents an emulsification time of less than 2 min, while the region along the vertical line represents an emulsification time greater than 2 min).

Self-microemulsification efficiency refers to the capacity of the SMEDDS to spontaneously form or disperse into a homogeneous microemulsion when the SMEDDS were added to water under mild agitation. The self-emulsifying equilibrium time and droplet size were used to assess self-microemulsification efficiency [21]. As shown in Fig 3, the highest content of oil phase in the SMEEDS is up to 40%, but the content of oil can not be too close to the critical point of the SMEEDS, because the change of the external environment may cause the SMEEDS critical point to shrink, which may lead to instability of the SMEEDS. In addition, when the content of oil was more than 15%, the emulsifying equilibrium time exceeded 2 min, which indicated that the self-emulsifying efficiency had decreased (Fig 3). Therefore, it is reasonable to fix the oil percentage at 15%.

The ratio of emulsifier to co-emulsifier was very effective for a stable and efficient SMEDDS formation. There was no significant difference for the solubility of Res in PEG400 and Cremophor RH40 *(P>0*.*05)* (Table 2). Therefore, the change of the content of emulsifier and co-emulsifier had no obvious effect on the drug loading of Res SMEDDS under the condition of fixed oil phase content, and the drug loading was approximately 9% (w/w). In order to prevent the precipitation of drugs in the storage process, the drug loading of Res SMEDDS was 5% (w/w) according to previous studies[15].

The change of the percentage of emulsifier (Cremophor RH40) and co-emulsifier (PEG400) will affect the efficiency of self-emulsification. With the increase of the percentage of co-emulsifier (PEG400), the emulsification time was significantly reduced *(P<0*.*05)* (Table 4). When the content of PEG400 in the SMEDDS increased from 5 to 20%, there were no obvious differences in droplet size. However, when the content of PEG400 increased from 20 to 35%, the droplet size increased from 27.69 nm to 812 nm. Excessive amount of co-emulsifier will cause the system to become unstable due to its high hydrophilicity, moreover, the droplet size will increase due to the expansion of the interfacial film[28, 29]. Therefore, 20% PEG400 should be chosen for the formulation.

**Table 4:** Design and result of the formulation optimization (at 37°C).

Prescription composition (W/W%)				Drug loading(mg/g)	Droplet size(nm)	Emulsifiction time (s)
No.	IPM	PEG400	Cremophor RH40			
1	15	5	80	86.07±1.25	27.85±0.91	454±28
2	15	10	75	86.47±1.82	23.86±0.85	320±35
3	15	15	70	87.31±2.25	27.61±1.53	245±20
4	15	20	65	90.40±1.11	27.69±0.87	117±23
5	15	25	60	94.00±1.85	59.21±2.57	83±17
6	15	30	55	94.20±1.52	193.8±8.22	44±5
7	15	35	50	87.32±1.72	812.8±19.26	15±3

Based on the above results, the SMEDDS formulation was a mixture of 15% (w/w) IPM, 65% (w/w) Cremophor RH40 and 20% (w/w) PEG400. The Res drug loading was 5% (w/w).

### Appearance

**Fig 4:**
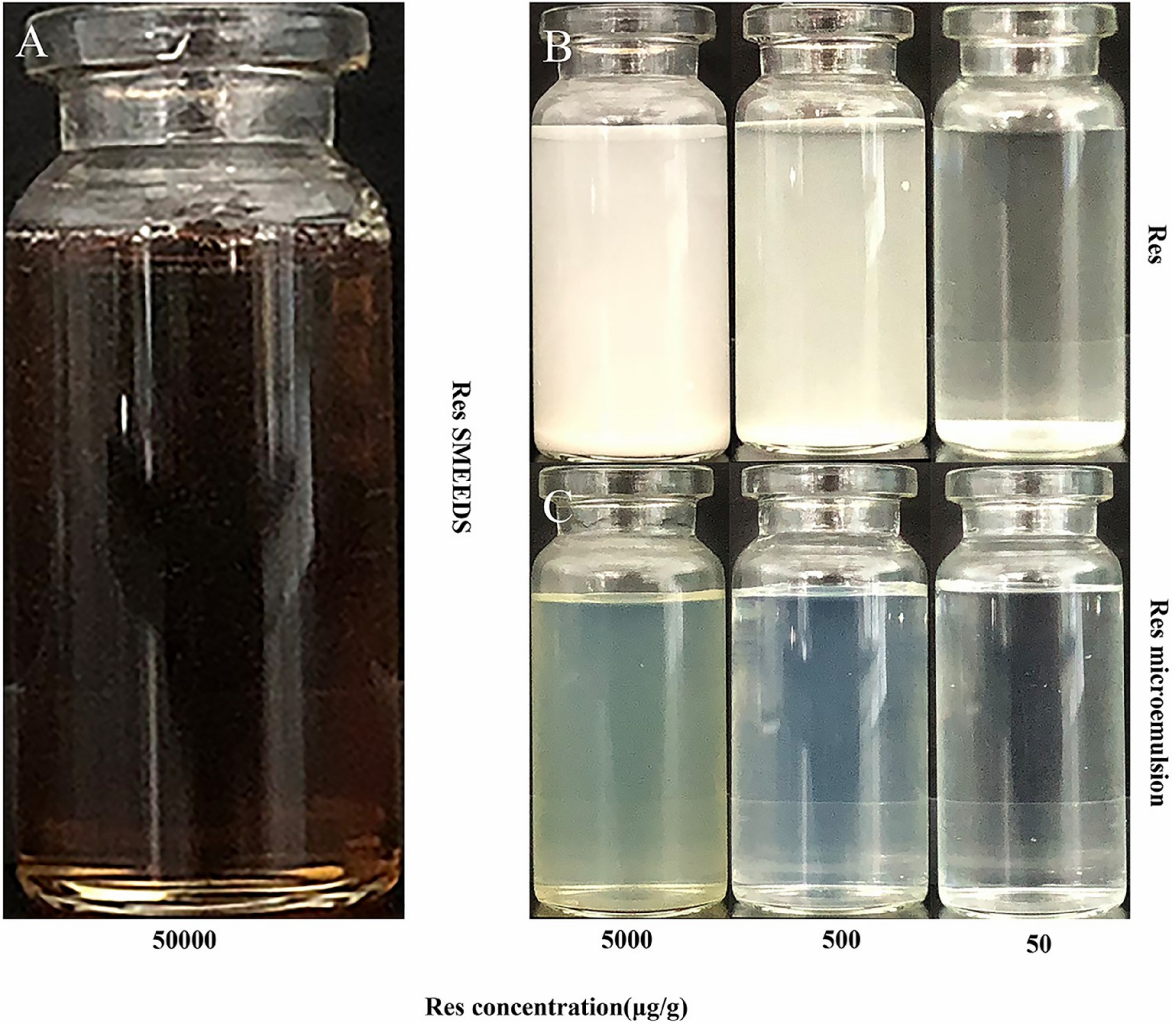
Appearance of resveratrol (Res) under different conditions. **(**A) Res SMEEDS; (B) Different concentrations of Res; (C) Different concentrations of Res microemulsion.

At room temperature, the Res SMEDDS (50 mg/g) was a viscous, transparent yellowish liquid. When the Res SMEDDS was serially tenfold diluted (10, 100 and 1000 times) with deionized water, the diluted solution becomes clear or slightly bluish. In contrast, under the same conditions, the same concentration of Res was suspended in water, even at a minimum concentration of 50 μg/g (Fig 4). The results indicated that the Res SMEDDS can improve the water solubility of Res, and the solubility of Res in SMEDDS was at least 1,000 times higher than that of the Res powder.

### Size and morphology

**Fig 5:**
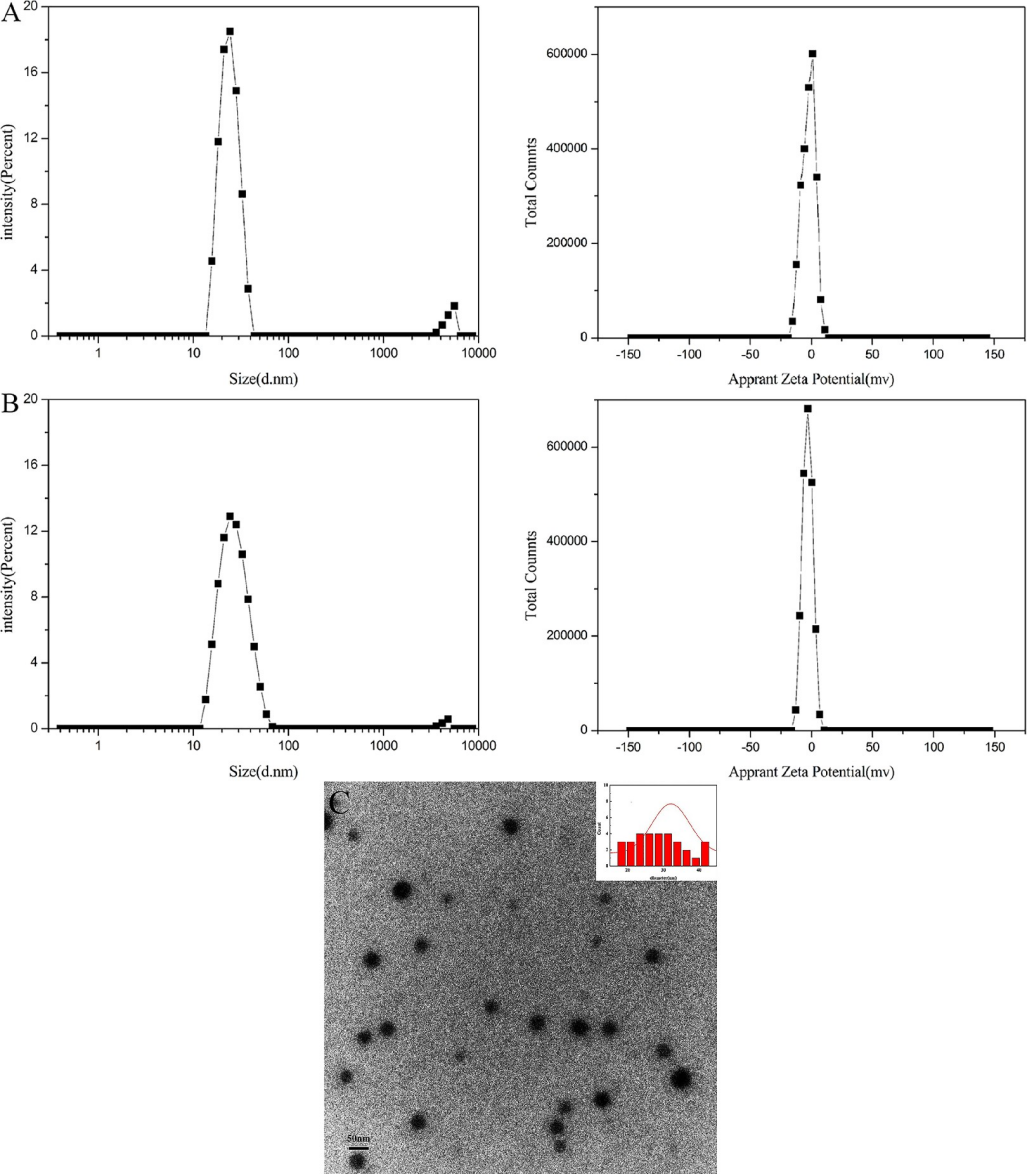
Drop size, zeta potential and TEM image of SMEDDS. (A) droplet size and zeta potential of the blank SMEDDS; (B) droplet size and zeta potential of the Res SMEDDS; (C) TEM image of the Res SMEDDS.

Droplet size is one of the most important parameters of microemulsion diluted from SMEDDS that affects the release rate of drug and drug stability. Smaller droplets have a greater interfacial area that significantly enhances the release rate of drug. After the Res SMEDDS was diluted 100 times with deionized water, the droplet size, zeta potential and TEM image were shown in Fig 5. Compared with the particle size of the blank nano-emulsion of 26.23±1.56nm, the particle size of the Res microemulsion was 28.00±1.67 nm, there was no significant difference (*P>0*.*05*). Therefore, 5% drug loading of Res had no significant effect on the particle size of the microemulsion. The PDI of the blank nano-emulsion and the Res nano-emulsion were 0.169 and 0.213, respectively, which indicated that Res SMEDDS exhibited good dispersion properties. The zeta potentials of the blank microemulsion and Res microemulsion were -2.18 mv and -3.25mv, and higher than that reported by Chen Y[15], which indicated that the Res microemulsion was more stable. Because charged ions on the surface of these nanoparticles prevented the aggregation and fusion of the nanoparticles by electrostatic repulsion[30].

Previous studies have shown that the droplet size of the Res microemulsion is generally in the range of 50–200 nm[15, 31, 32], while the Res microemulsion prepared in this work had a smaller droplet size with an average droplet size of 28.00±1.67 nm, which was smaller that those that have been reported by Chen Y [15]. The smaller the particle size of the microemulsion is, the easier it is to be absorbed [33], therefore, Res SMEDDS prepared in this work may be exhibit better bioavailability. In addition, the TEM image revealed that the morphology of the Res emulsion was a regular circle with good dispersibility, which is consistent with the results of Chen Y[15]. Statistical analysis of the droplet size of the droplets in the TEM image with the NanoMeasure software also revealed that the average droplet size of the Res SMEDDS was about 28.81 nm, which was consistent with the droplet size measurement results.

### *In vitro* stability of Res SMEDDS

The effect of the temperature on the stability parameters of the SMEEDS is shown in Table 5. There was no significant difference in the appearance, drug loading and droplet size of the Res SMEDDS at 4 and 40°C in the dark for 10 days *(P>0*.*05)*, which demonstrated that the Res SMEDDS had excellent stability. At the temperature of 60°C for 10 days, the appearance color of the Res SMEDDS became darker. The reason is that the cloud point of Cremophor RH40 is 44°C,when the temperature is higher than the cloud point of Cremophor RH40, irreversible phase separation occurs, which lead to become a darker solution. In addition, the Res content was reduced due to the instability of Res at 60°C[34], The effect of exposure to light on the stability of the SMEDDS is shown in Table 6, under long-term illumination (4500Lx), the Res drug loading of the Res SMEDDS decreased on the 10th day due to the light instability of Res[35]. Therefore, the Res SMEDDS should be stored in the dark below 40°C.

**Table 5:** Influence of the temperature on the stability of the Res SMEDDS (n = 3).

T/°C	Days	Appearance	D (nm)	Drug loading (mg/g)
4	0	Yellow and transparent	28.21±0.94	51.95±0.32
4	5	Yellow and transparent	27.61±0.88	51.20±0.22
4	10	Yellow and transparent	25.74±1.21	50.15±0.18
40	0	Yellow and transparent	28.21±0.94	51.95±0.32
40	5	Yellow and transparent	26.75±1.36	50.44±0.23
40	10	Yellow and transparent	25.03±1.13	52.17±0.21
60	0	Yellow and transparent	28.21±0.94	51.95±0.32
60	5	Tan and transparent	27.69±0.87	48.95±0.26
60	10	Tan and transparent	27.21±0.61	45.94±0.18

**Table 6:** Stability of the Res SMEDDS under illumination (4500Lx) condition (n = 3).

Illumination time/d	Appearance	D (nm)	Drug loading (mg/g)
0	Yellow and transparent	28.21±0.94	51.95±0.32
5	Yellow and transparent	26.81±0.18	50.50±0.26
10	Yellow and transparent	27.21±0.16	46.80±0.35

### *In vitro* elution degree

**Fig 6:**
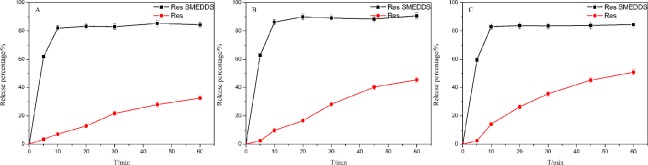
Release percentage of the Res SMEDDS and Res powder at different pH values. (A) The pH of the dissolution medium is 1.2; (B) The pH of the dissolution medium is 6.8; (C) The pH of the dissolution medium is 7.4.

The release profiles of Res powder drug and Res SMEDDS at different pH values were shown in Fig 6. The release percentage of Res from Res SMEDDS was more than 80% within 10 minutes, whereas the highest release percentage of Res powder drug was less than 55% within 60 minutes at the three different pH values. Statistically significant differences were observed between the two formulations at the same pH *(P<0*.*05)*. The free energy required to form microemulsion is very low, spontaneous formation of microemulsion accelerate the dissolution of drug. In addition, smaller droplet size and larger interfacial area also advantageously increase the drug release rate[36], therefore, Res SMEDDS was able to enhance the *in vitro* release of Res.

As shown in Fig 6, pH values of media had no effect on the *in vitro* release of Res from Res SMEDDS *(P>0*.*05)*, which is consistent with release properties of phenol compounds Gingerol-SMEDDS reported by Xu Y et al[37]. However, the cumulative percentages of the Res powder at different pH values were 32.53±1.83 (pH = 1.2), 45.40±1.91 (pH = 6.8) and 50.87±1.87 (pH = 7.4), which was significant difference at the three different pH values *(P<0*.*05)*.Release of Res powder is dependent on its solubility, the solubility of Res is sensitive to pH[38]. In addition, the major intermolecular interactions of Res powder are through hydrogen bonds, Each of the three oxygen atoms in the hydroxyl groups participates in two hydrogen bond intermolecular interactions[39], breakdown of intermolecular hydrogen bond may be promote dissolution of Res due to weak basic medium. Therefore, release of Res powder was affected by pH values of media.

In addition, the particle size of the Res SMEDDS did not change significantly and the distribution was uniform at different pH values *(P>0*.*05)* (Table 7), which indicated that the Res SMEDDS was stable, and Res release of Res SMEDDS was unaffected by pH values of media. Because Res in Res SMEDDS was encapsulated and mainly located in the core of core/shell microemulsions[40], which led to stable release of Res from Res SMEDDS and no significant change of nanoparticle size and distribution.

**Table 7:** Particle Size and Distribution Index at Different pH values.

	D (nm)	PDI
pH = 1.2	25.11±0.23	0.115±0.012
pH = 6.8	25.42±0.34	0.099±0.013
pH = 7.4	26.43±0.18	0.128±0.018

These results suggested that the *in vitro* release of Res from Res SMEDDS was faster that of Res powder and unaffected by pH value of media.

### *In vitro* antioxidant

**Fig 7:**
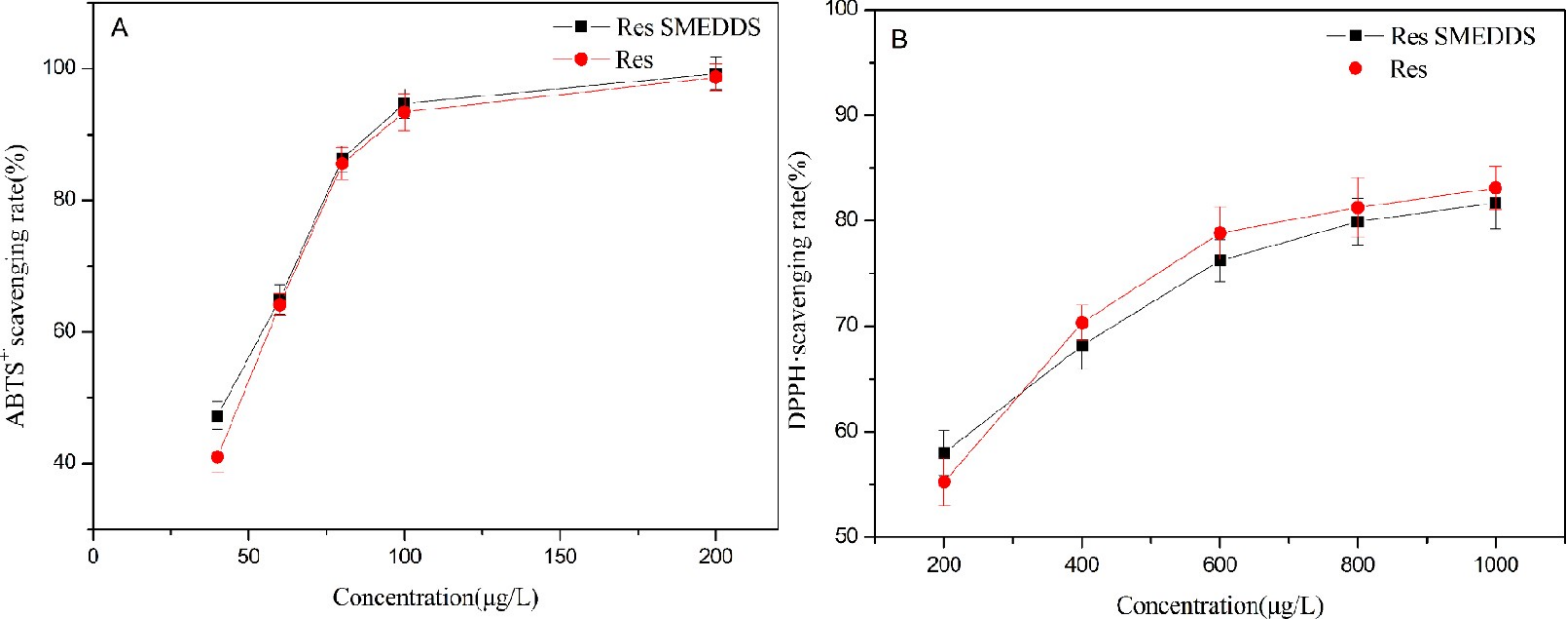
Radical scavenging rate of the Res SMEDDS and Res powder. (A) ABTS+• free radical scavenging rates; (B) DPPH· free radical scavenging rates.

There are many methods to evaluate the antioxidant activity of microemulsions *in vitro*, among which the DPPH free radical scavenging method and the ABTS free radical scavenging method are commonly used. The results revealed that the DPPH· and ABTS^+·^scavenging rate increased gradually with the increase of the Res concentration (Fig 7), which indicated that ABTS^+·^ and DPPH·scavenging capacity of Res exhibited a certain dose-response relationship. However, there was no significant difference in the DPPH· and ABTS^+·^ free radical scavenging rates between the Res SMEDDS and Res powder at the same Res concentration *(P>0*.*05)*. This means that the Res SMEDDS and Res powder had similar scavenging activity.

### Cytotoxicity

PC12 cells were treated with different concentrations of Res powder and Res SMEDDS,and their effects on the RGR of PC12 cells and toxicity grades were evaluated based on the detected OD(S1 File).

The MTT assay showed that the RGR of the cells decreased with the increase of drug concentration. When the drug concentration exceeded 20μM, compared with Res powder, the RGR of Res SMEDDS was smaller and statistically significant *(P <0*.*05)* (Table 8), which demonstrated that Res SMEDDS was slightly higher cytotoxic to PC12 cells than that of Res powder due to slightly toxic of the components in SMEDDS. However, according to the RGR classification of ISO 10993-5-2009 standard, the cytotoxicity of each concentration group of Res SMEDDS belongs to "1" grade(Table 8), therefore, Res SMEDDS with a concentration below 100 μM was safe[41].

**Table 8:** Toxicity of Res SMEDDS and Res to PC12 cells.

Groups	Concentration/μM	RGR/%	grades
Control	0	100	0
Res	10	99.69±2.03	1
	20	99.09±2.86	1
	50	92.71±3.31	1
	100	88.40±1.12	1
Res SMEDDS	10	94.71±3.23	1
	20	91.73±4.23	1
	50	83.31±1.08	1
	100	78.28±3.98	1

## Conclusion

A fat-soluble drug Res is insufficiently absorbed by the human body due to its poor solubility. Accordingly, formulation strategies for enhancing solubility and dissolution rate of poorly water-soluble drugs are developed to improve the oral bioavailability. In this work, the developed Res SMEDDS system, composed of IPM (15%), PEG400 (20%), and Cremophor RH40 (65%), was found to significantly enhance the solubility of Res. Res SMEDDS needed to keep under 40°C and avoid light exposure. The *in vitro* release of Res from Res SMEDDS was significantly faster that of Res powder and unaffected by pH value of media. There was no significant change for antioxidant activities of Res in presence of Res SMEDDS compared to Res powder. Res SMEDDS at the concentration of less than 100 μM was safe. These results demonstrated the potential use of Res SMEDDS for oral administration of Res.

## Supporting information

S1 File Data and sources. Treatment of PC12 cells with OD at 570 nm with different concentrations of Res powder and Res SMEDDS.
(ZIP)
